# Troponin I and T in relation to cardiac injury detected with electrocardiography in a population-based cohort - The Maastricht Study

**DOI:** 10.1038/s41598-017-06978-3

**Published:** 2017-07-26

**Authors:** Dorien M. Kimenai, Remy J. H. Martens, Jeroen P. Kooman, Coen D. A. Stehouwer, Frans E. S. Tan, Nicolaas C. Schaper, Pieter C. Dagnelie, Miranda T. Schram, Carla J. H. van der Kallen, Simone J. S. Sep, Jeroen D. E. van Suijlen, Abraham A. Kroon, Otto Bekers, Marja P. van Dieijen-Visser, Ronald M. A. Henry, Steven J. R. Meex

**Affiliations:** 1 Department of Clinical Chemistry, Central Diagnostic Laboratory, Maastricht University Medical Center+ (MUMC+), Maastricht, The Netherlands; 2 CARIM School for Cardiovascular Diseases, Maastricht University, Maastricht, The Netherlands; 3 Department of Internal Medicine, Division of Nephrology, MUMC+, Maastricht, The Netherlands; 4 NUTRIM School of Nutrition and Translational Research in Metabolism, Maastricht University, Maastricht, The Netherlands; 5 Department of Internal Medicine, MUMC+, Maastricht, The Netherlands; 6 CAPHRI School for Public Health and Primary Care, Maastricht University, Maastricht, The Netherlands; 7 Department of Methodology and Statistics, Maastricht University, Maastricht, The Netherlands; 8 Department of Epidemiology, MUMC+, Maastricht, The Netherlands; 9 Heart and Vascular Centre, MUMC+, Maastricht, The Netherlands; 10 Department of Clinical Chemistry, Gelre Hospital, Apeldoorn, The Netherlands

## Abstract

Interest in high-sensitivity cardiac troponin I(hs-cTnI) and T(hs-cTnT) has expanded from acute cardiac care to cardiovascular disease(CVD) risk stratification. Whether hs-cTnI and hs-cTnT are interchangeable in the ambulant setting is largely unexplored. Cardiac injury is a mechanism that may underlie the associations between troponin levels and mortality in the general population. In the population-based Maastricht Study, we assessed the correlation and concordance between hs-cTnI and hs-cTnT. Multiple regression analyses were conducted to assess the association of hs-cTnI and hs-cTnT with electrocardiographic (ECG) changes indicative of cardiac abnormalities. In 3016 eligible individuals(mean age,60 ± 8years;50.6%,men) we found a modest correlation between hs-cTnI and hs-cTnT(r = 0.585). After multiple adjustment, the association with ECG changes indicative of cardiac abnormalities was similar for both hs-cTn assays(OR,hs-cTnI:1.72,95%CI:1.40-2.10;OR,hs-cTnT:1.60,95%CI:1.22–2.11). The concordance of dichotomized hs-cTnI and hs-cTnT was κ = 0.397(≥sex-specific 75^th^ percentile). Isolated high levels of hs-cTnI were associated with ECG changes indicative of cardiac abnormalities(OR:1.93,95%CI:1.01–3.68), whereas isolated high levels of hs-cTnT were not(OR:1.07,95%CI:0.49–2.31). In conclusion, there is a moderate correlation and limited concordance between hs-cTnI and hs-cTnT under non-acute conditions. These data suggest that associations of hs-cTnI and hs-cTnT with cardiac injury detected by ECG are driven by different mechanisms. This information may benefit future development of CVD risk stratification algorithms.

## Introduction

The cardiac troponins I and T are the preferred biomarkers for diagnosing acute myocardial infarction (AMI)^1^. Whereas conventional troponin assays were limited to the detection of plasma troponin concentrations that are typically seen in the setting of acute cardiac injury, high-sensitivity assays can detect cardiac troponin concentrations below the clinical cut-off levels^2, 3^. In parallel with this technical progress, interest in cardiac troponin has expanded from acute cardiac care to cardiovascular disease (CVD) risk stratification. Indeed, epidemiological studies have demonstrated that basal levels of high-sensitivity cardiac troponin I (hs-cTnI) and T (hs-cTnT) are invariably associated with cardiac morbidity and mortality, even in apparently healthy individuals^4–17^.

Whether hs-cTnI and hs-cTnT are interchangeable in CVD risk stratification algorithms is largely unexplored. An important issue here is the fact that, although during and after AMI, hs-cTnI and hs-cTnT levels in blood are strongly correlated (reported r = 0.85)^13^, and carry equivalent diagnostic value^18^, they not do so under non-acute conditions (reported r = 0.44–0.70)^13, 19–21^. This surprising discrepancy may suggest different mechanisms of release or clearance for troponin I and T in the chronic setting, but more importantly, it raises the hypothesis that associations of cardiac troponin I and T with cardiac morbidity and mortality may be different.

Cardiac injury is a plausible mechanism that may underlie the associations between basal troponin levels and cardiac morbidity and mortality in the general population, and more insight in the relationship between cardiac troponin I and T with cardiac injury can provide useful data for cardiovascular risk prediction^22^. Electrocardiography (ECG) changes are possibly indicative of cardiac injury (e.g., cardiac ischemia). To test the hypothesis that the modest hs-cTnI-hs-cTnT correlation also translates into different associations with cardiac injury, we directly compared the associations of hs-cTnI and hs-cTnT with cardiac injury detected with ECG in a population-based cohort. In addition, we explored whether hs-cTnI and hs-cTnT provide interchangeable information, in terms of their association with ECG changes indicative of cardiac abnormalities.

## Results

### Study population

A total of 3016 eligible participants from The Maastricht Study cohort were included in the present study (see Supplementary Fig. S1). Clinical characteristics of the total study population, and stratified by the presence of ECG changes indicative of cardiac abnormalities, are shown in Table 1. The overall proportion of participants with detectable hs-cTnT levels (Limit of Detection (LoD) ≥5 ng/L) was 54%. The overall proportion of detectable hs-cTnI levels ranged from 51% (LoD ≥1.9 ng/L) *to* 83% (LoD ≥1.1 ng/L). The overall proportion participants with hs-cTn levels above the 10% coefficient of variation (CV) for hs-cTnT and hs-cTnI were 6% and 10%, respectively. CVD risk factors were more prevalent in participants with ECG changes indicative of cardiac abnormalities as compared with participants without ECG changes indicative of cardiac abnormalities (Table 1). The number (%) of Minnesota coding categories of ECG abnormalities of the total study population are shown in Supplementary Table S1.

**Table 1:** Clinical characteristics of the total study population and stratified by the presence of ECG changes indicative of cardiac abnormalities.

Variable	Total study population (n = 3016)	No ECG changes indicative of cardiac abnormalities (n = 2923)	ECG changes indicative of cardiac abnormalities (n = 93)
age (years)	60 (8)	60 (8)	63 (8)
sex (male)	1526 (50.6%)	1479 (50.6%)	47 (50.5%)
BMI (kg/m^2^)	26.9 (4.5)	26.9 (4.5)	28.4 (5.0)
**eGFR**			
<60 mL/min/1.73 m^2^	109 (3.6%)	103 (3.5%)	6 (6.5%)
60 -<90 mL/min/1.73 m^2^	1438 (47.7%)	1393 (47.7%)	45 (48.4%)
≥90 mL/min/1.73 m^2^	1469 (48.7%)	1427 (48.8%)	42 (45.2%)
**glucose metabolism**			
NGM	1776 (58.9%)	1739 (59.5%)	37 (39.8%)
IFG	129 (4.3%)	125 (4.3%)	4 (4.3%)
IGT	330 (10.9%)	312 (10.7%)	18 (19.4%)
T2DM	781 (25.9%)	747 (25.6%)	34 (36.6%)
office BP, systolic (mmHg)	135 (18)	134 (18)	140 (17)
office BP, diastolic (mmHg)	76 (10)	76 (10)	77 (11)
hypertension (yes)	1628 (54.0%)	1557 (53.3%)	71 (76.3%)
antihypertensive medication (yes)	1108 (36.7%)	1056 (36.1%)	52 (55.9%)
**smoking behavior**			
never	1090 (36.1%)	1055 (36.1%)	35 (37.6%)
former	1525 (50.6%)	1476 (50.5%)	49 (52.7%)
current	401 (13.3%)	392 (13.4%)	9 (9.7%)
total-to-HDL cholesterol ratio	3.5 (2.8–4.3)	3.5 (2.8–4.3)	3.4 (2.7–4.4)
triglycerides (mmol/L)	1.2 (0.9–1.7)	1.2 (0.9–1.7)	1.3 (0.9–1.6)
lipid-modifying medication (yes)	992 (32.9%)	943 (32.3%)	49 (52.7%)
hs-cTnI (ng/L)	1.9 (1.2–3.0)	1.8 (1.2–2.9)	2.8 (1.6–4.9)
hs-cTnT (ng/L)	5.3 (3.8–7.7)	5.3 (3.7–7.6)	7.0 (4.7–11.3)

Continuous variables are expressed as mean (SD) or median (IQR) depending on their distribution. Categorical data are reported as n (%). Abbreviations: BP, blood pressure; BMI, Body Mass Index; eGFR, estimated Glomerular Filtration Rate; HDL, high-density lipoprotein; hs-cTnI, high-sensitivity cardiac troponin I; hs-cTnT, high-sensitivity cardiac troponin T; NGM, normal glucose metabolism; IFG, impaired fasting glucose; IGT, impaired glucose tolerance; T2DM, type 2 diabetes mellitus.

### Modest correlation between hs-cTnI and hs-cTnT

**Figure 1:**
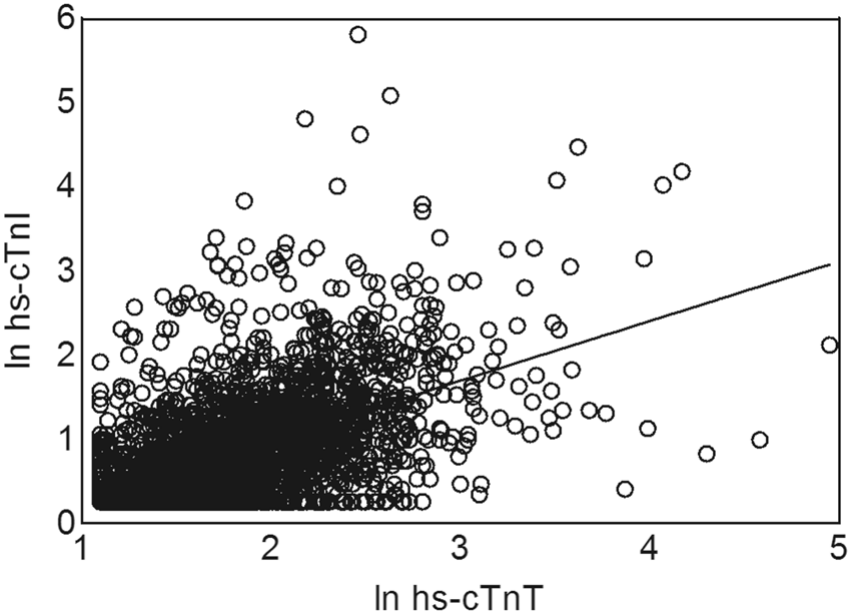
Correlation between natural log transformed hs-cTnI and natural log transformed hs-cTnT (β = 0.465, 95% CI 0.442–0.488, *p* < 0.001, R^2^ = 0.343). Abbreviations: hs-cTnI, high-sensitivity cardiac troponin I; hs-cTnT, high-sensitivity cardiac troponin T; ln, natural log transformed.

In contrast to the acute cardiac care setting where troponin I and T are interchangeable^18^, a remarkably modest correlation was found between hs-cTnI and hs-cTnT in these ambulatory participants (r = 0.585, 95% CI 0.562–0.608, *p* < 0.001, Fig. 1). The modest correlation was not due to discrepant sensitivities of hs-cTn assays. In the analyses we corrected for the proportion of participants below the LoB, and even after excluding participants with hs-cTn below the LoD (LoD hs-cTnI: 1.9 ng/L, LoD hs-cTnT: 5 ng/L), which equalized the proportion of participants with detectable hs-cTnT levels to those of the hs-cTnI assay, the correlation did not improve (r = 0.373, 95% CI 0.322–0.420, *p* < 0.001, n = 1180).

### Hs-cTnI and hs-cTnT are independently associated with cardiac injury detected with ECG

To test whether the modest hs-cTnI-hs-cTnT correlation also translated into different associations with cardiac injury, we directly compared the associations of hs-cTnI and hs-cTnT with cardiac injury detected with ECG. Univariable associations of hs-cTnI and hs-cTnT with ECG changes indicative of cardiac abnormalities were statistically significant and numerically similar (Table 2, Model 1). Sequential adjustments for demographic variables, eGFR and traditional CVD risk factors did not abrogate the similar associations of both hs-cTn with ECG changes indicative of cardiac abnormalities (odds ratio (OR) per 1-SD higher ln hs-cTnI: 1.72, 95% CI 1.40–2.10; OR per 1-SD higher ln hs-cTnT: 1.60, 95% CI 1.22–2.11) (Table 2, Model 4 A). Both hs-cTnI and hs-cTnT showed no significant interaction with eGFR on the association with ECG changes indicative of cardiac abnormalities (hs-cTnI*eGFR, *p*_interaction_ = 0.991; hs-cTnT*eGFR, *p*_interaction_ = 0.409). Results were robust in sensitivity analyses, of which values below the LoD, 20% CV, 10% CV, and LoQ were set equal to these lower limits of measurements (see Supplementary Table S2). However, the association between hs-cTnT and ECG changes indicative of cardiac abnormalities remained not statistically significant when hs-cTnT values below 13 ng/L were set at 13 ng/L (10% CV, LoQ) (see Supplementary Table S2). Results were not materially altered with a more liberal definition of ECG changes indicative of cardiac abnormalities (definition included also borderline Q/QS waves, or ST-segment abnormalities accompanied by abnormal T-waves) (see Supplementary Table S3).

**Table 2:** Associations of hs-cTnI and hs-cTnT with ECG changes indicative of cardiac abnormalities.

	Hs-cTnI		Hs-cTnT	
	ln, 1-SD increase		ln, 1-SD increase	
	OR (95% CI)	*p*-value	OR (95% CI)	*p*-value
model 1	1.72 (1.45–2.05)	<0.001	1.67 (1.36–2.05)	<0.001
model 2	1.72 (1.42–2.09)	<0.001	1.56 (1.22–2.01)	0.001
model 3	1.76 (1.45–2.14)	<0.001	1.67 (1.28–2.18)	<0.001
model 4A	1.72 (1.40–2.10)	<0.001	1.60 (1.22–2.11)	0.001
model 4B	1.89 (1.53–2.34)	<0.001	1.65 (1.24–2.20)	0.001

Model 1: crude model; model 2: model 1+ sex, age, glucose metabolism status; model 3: model 2+ eGFR; model 4A: model 3+ smoking behavior, total-to-HDL cholesterol ratio, triglyceride levels, lipid-modifying medication, office systolic blood pressure, antihypertensive medication, waist-to-hip ratio, alcohol consumption, educational level; model 4B: model 4A with replacement of office systolic blood pressure by 24 h average ambulatory systolic blood pressure. Abbreviations: ECG, electrocardiographic; eGFR, estimated Glomerular Filtration Rate; HDL, high-density lipoprotein; hs-cTnI, high-sensitivity cardiac troponin I; hs-cTnT, high-sensitivity cardiac troponin T.

### Discordance of hs-cTnI and hs-cTnT assays

**Figure 2:**
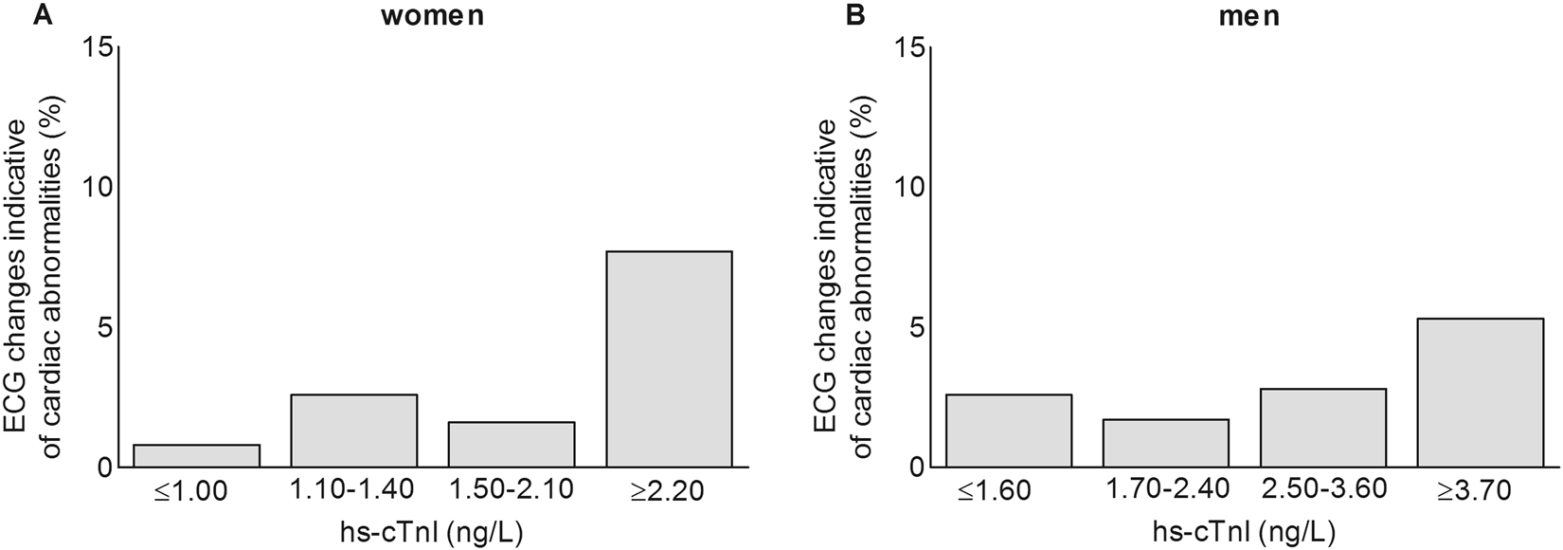
Prevalence of ECG changes indicative of cardiac abnormalities per hs-cTnI quartile, stratified by sex (women; panel A: men; panel B). Abbreviations: ECG, electrocardiographic; hs-cTnI, high-sensitivity cardiac troponin I.

Study participants were classified into four groups on the basis of dichotomized hs-cTn concentrations, divided at the sex-specific 75^th^ percentile (hs-cTnI, women: 2.20 ng/L; hs-cTnI, men: 3.70 ng/L; hs-cTnT, women: 5.55 ng/L; hs-cTnT, men: 9.36 ng/L). Dichotomization at the 75^th^ percentile was based on a troponin I threshold effect corresponding to the upper quartile. This threshold effect for troponin I was first described in relation to cardiovascular death and heart failure^19^ and now further extended in this study in relation to the presence of ECG changes indicative of cardiac abnormalities (Fig. 2). Clinical characteristics of the groups based on sex-specific 75^th^ percentiles (and sex-specific 99^th^ percentiles) per hs-cTn assay are shown in Supplementary Table S4 and S5. The concordance between the two assays was statistically significant but rather weak (Cohen’s kappa (κ) 0.397; 95% CI 0.359–0.434), with 22.4% of participants having a discordant classification (Table 3).

**Table 3:** Concordance of hs-cTnI and hs-cTnT according to sex-specific 75^th^ percentile thresholds.

		Hs-cTnT ≥ sex-specific 75^th^ percentile		
		No	Yes	Total
Hs-cTnI ≥ sex-specific 75^th^ percentile	No	1934 (64.1%)	344 (11.4%)	2278 (75.5%)
	Yes	332 (11.0%)	406 (13.5%)	738 (24.5%)
	Total	2266 (75.1%)	750 (24.9%)	3016 (100%)

Data are reported as n (%). Cohen’s κ = 0.397 (95% CI 0.359-0.434). Four groups were classified according to sex-specific 75^th^ percentiles of hs-cTnI and hs-cTnT (hs-cTnI, women: 2.20 ng/L; hs-cTnI, men: 3.70 ng/L;hs-cTnT, women: 5.55 ng/L; hs-cTnT, men: 9.36 ng/L). Abbreviations: hs-cTnI, high-sensitivity cardiac troponin I; hs-cTnT, high-sensitivity cardiac troponin T.

**Figure 3:**
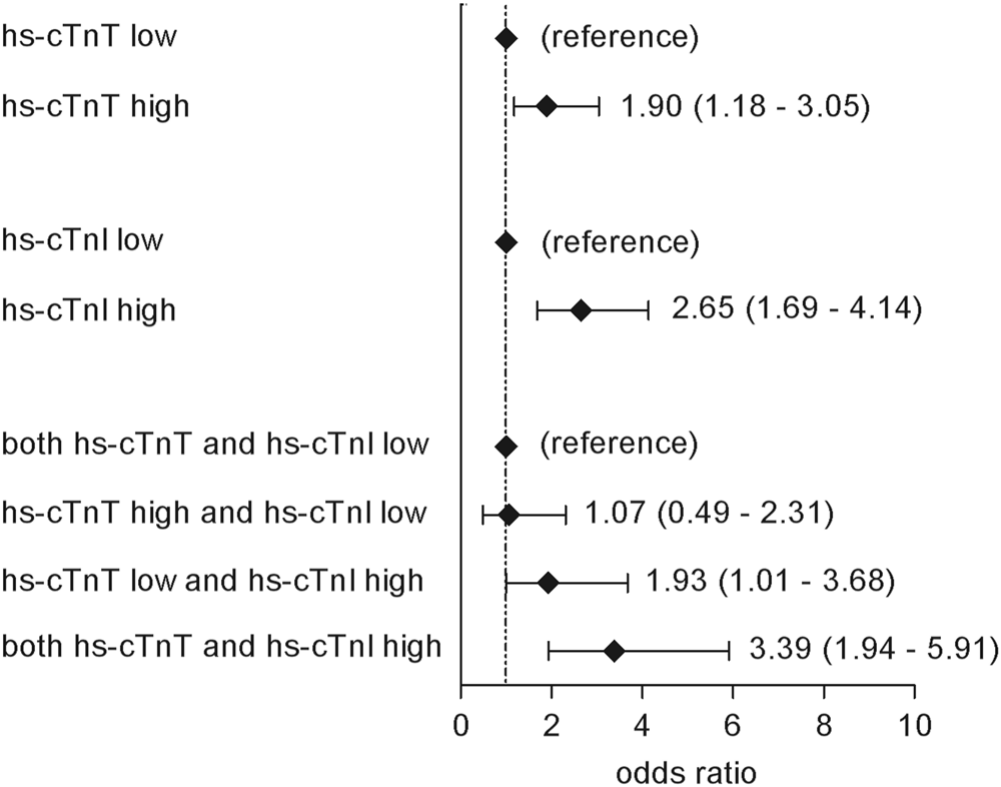
Associations between combined hs-cTn categories and ECG changes indicative of cardiac abnormalities. Definitions of “low” and “high” hs-cTn categories were based on the sex-specific 75^th^ percentiles of hs-cTnI and hs-cTnT (hs-cTnI, women: 2.20 ng/L; hs-cTnI, men: 3.70 ng/L; hs-cTnT, women: 5.55 ng/L; hs-cTnT, men: 9.36 ng/L). Category “low” included participants with hs-cTn levels <the sex-specific 75^th^ percentile and category “high” included participants ≥the sex-specific 75^th^ percentile. Model was adjusted for sex, age, glucose metabolism status, eGFR, smoking behavior, total-to-HDL cholesterol ratio, triglyceride levels, lipid-modifying medication, office systolic blood pressure, antihypertensive medication, waist-to-hip ratio, alcohol consumption and educational level. Abbreviations: ECG, electrocardiographic; eGFR, estimated Glomerular Filtration Rate; HDL, high-density lipoprotein; hs-cTnI, high-sensitivity cardiac troponin I; hs-cTnT, high-sensitivity cardiac troponin T.

We then assessed the associations of dichotomized hs-cTn concentrations with ECG changes indicative of cardiac abnormalities. Both high levels of hs-cTnI and hs-cTnT were strongly associated with ECG changes indicative of cardiac abnormalities (Fig. 3). The strongest association with ECG changes indicative of cardiac abnormalities was observed for participants who had high levels of both hs-cTnI and hs-cTnT (“both hs-cTnT and hs-cTnI high” as compared with the reference category “both hs-cTnT and hs-cTnI low”, OR 3.39, 95% CI 1.94–5.91, *p* < 0.001) (Fig. 3). As compared with the category “both hs-cTnT and hs-cTnI low”, isolated high levels of troponin I were associated with ECG changes indicative of cardiac abnormalities (OR 1.93, 95% CI 1.01–3.68, *p* = 0.046), whereas isolated high levels of troponin T were not (OR 1.07, 95% CI 0.49–2.31, *p* = 0.869) (Fig. 3). These results were robust in a sensitivity analysis in which we used a more liberal definition of ECG changes indicative of cardiac abnormalities (see Supplementary Fig. S2), and when we applied a range of alternative percentile cut-off points (percentile 66^th^ to percentile 90^th^) to dichotomize cardiac troponin levels (see Supplementary Table S6).

### Clinical characteristics of discordantly classified participants

In an exploratory analysis to further investigate the discordance between both hs-cTn assays, we compared clinical parameters of concordant and discordantly classified participants. Discordantly classified participants differed most markedly in estimated glomerular filtration rate (eGFR) and the prevalence of type 2 diabetes mellitus (T2DM): prevalence of eGFR <60 mL/min/1.73 m^2^ was 7.6% in participants with isolated high hs-cTnT levels compared with 1.5% for participants with isolated high hs-cTnI levels (see Supplementary Table S7). Prevalence of T2DM was 42.7% *vs* 20.5% in participants with isolated high hs-cTnT levels compared with participants with isolated high hs-cTnI levels, respectively.

## Discussion

This study reports four major findings. 1) Hs-cTnI and hs-cTnT were only moderately correlated in the general population. 2) There was substantial discordance between hs-cTnI and hs-cTnT under non-acute conditions. 3) Higher hs-cTnI and hs-cTnT were similarly associated with ECG changes indicative of cardiac abnormalities independent of demographic variables, eGFR and CVD risk factors, and 4) isolated high levels of troponin I, but not isolated high levels of troponin T, were associated with ECG changes indicative of cardiac abnormalities, independent of the same risk factors as under 3.

In contrast to the setting of acute coronary syndrome, where hs-cTnI and hs-cTnT are strongly correlated^13^ and are considered diagnostically interchangeable^18^, recent data suggest a much lower correlation under non-acute conditions such as atrium fibrillation^20^, stable coronary artery disease^19^, and chronic kidney disease^21^ (see Supplementary Table S8). This may reflect different mechanisms that drive troponin I and T elevations in the chronic setting. The present results corroborate and extend previously reported data by showing substantial discordance between troponin I and T elevations in a population-based cohort. This study focused on the relationship with cardiac injury, which is a possible mechanism for troponin I and T elevations in the ambulant setting. We found that isolated elevations of troponin I, but not isolated troponin T levels, were associated with ECG changes indicative of cardiac abnormalities. This would suggest that the similar strength of the association for hs-cTnI and hs-cTnT with ECG changes indicative of cardiac abnormalities are due to different underlying mechanisms. Hs-cTnI and hs-cTnT may therefore not be interchangeable as predictor for cardiovascular events and mortality in the ambulant setting, or on the other hand, they may even be complementary to each other. Unraveling the discrepancies between both hs-cTn may contribute to the development of CVD risk stratification algorithms that comprise cardiac biomarkers.

The source of discordant hs-cTnI and hs-cTnT measurements can be analytical or biological. Differences in imprecision between hs-cTnI and hs-cTnT are an unlikely source of the modest correlation between hs-cTnI and hs-cTnT: when participants below an LoD of 1.9 ng/L were excluded, which brought the proportion of participants with measurable troponin I concentrations to a similar percentage as the troponin T assay (hs-cTnI 51%, hs-cTnT 54%), the correlation between troponin I and T became even weaker (r = 0.373 *vs* r = 0.585).

Lack of analytical specificity due to interference of heterophilic or other antibodies is also unlikely to account for the substantial level of discordance and modest correlation between hs-cTnI and hs-cTnT. Hence, we speculate that true biological differences in release and/or elimination underlie the moderate correlation between hs-cTn assays in the chronic setting. We further explored which variables were associated with the distinct distribution patterns of troponin T and I, and found that a reduced eGFR and T2DM were more prevalent in participants with isolated elevations of troponin T, which is in line with the findings of Hijazi *et al*.^20^. Contrary to their findings, we observed no differences in age and proportion of men, but this is probably due to the age limit of 75 years of The Maastricht Study and the fact that we used sex-specific thresholds in our definition of elevated hs-cTn levels, while they did not. Whether these particular traits are mechanistically involved in distinct release and/or clearance patterns of troponin T and I is unknown. In this regard, previous studies have shown a stronger association of hs-cTnT than hs-cTnI with eGFR^14, 21, 23–25^, and our results expand these data by showing a higher prevalence of reduced eGFR in participants with isolated high hs-cTnT levels.

This study has several limitations. 1) The cross-sectional nature of our study precludes assessment of longitudinal associations of hs-cTnI and hs-cTnT with incident cardiac morbidity and mortality. Although the results are in line with longitudinal associations observed in a cohort with atrium fibrillation^20^, and extend this phenomenon to individuals from a population-based cohort, the next relevant step should be investigating whether troponin I and T are interchangeable predictors for cardiovascular morbidity and mortality in the general population. 2) Only one hs-cTnI assay was studied (Abbott), and the lack of standardization of hs-cTnI assays precludes extrapolation of our data to other troponin I assays. 3) The study participants were mainly of Caucasian origin, limiting generalizability to other ethnic groups. 4) The definition of cardiac injury is based on ECG data only, without additional information on cardiac ischemia from coronary imaging or echocardiography. Imaging modalities were not yet available in the current dataset. Although the selected ECG criteria indicate the relative likelihood of signs of cardiac ischemia, ECG abnormalities due to for e.g. left ventricular hypertrophy, dilated cardiomyopathy or amyloid cardiomyopathy cannot be excluded. 5) Although hs-cTnI and hs-cTnT were measured with high-sensitivity assays, concentrations were still below the LoB and LoD in a substantial number of participants. 6) To obtain a direct comparison between hs-cTnI and hs-cTnT assay, we corrected in the analysis for assay performance by adjustment of lower limits measurements. However, the lower sensitivity of hs-cTnT assay may have limited to show the association with hs-cTnT levels and ECG changes indicative of cardiac abnormalities.

In conclusion, this study showed a moderate correlation and limited concordance between the hs-cTnI and hs-cTnT assays. We showed that isolated high troponin I levels, but not isolated high troponin T levels, were independently associated with cardiac injury detected with ECG under non-acute conditions. These data suggest that observed associations of hs-cTnI and hs-cTnT with ECG changes indicative of cardiac abnormalities are driven by different mechanisms. This information may benefit future development of CVD risk stratification algorithms, using information from high–sensitivity cardiac troponin I and T assays.

## Methods

### Study population

We used data from The Maastricht Study, an ongoing observational prospective population-based cohort study. The rationale and methodology have been described previously^26^. In brief, the study focuses on the etiology, pathophysiology, complications and comorbidities of T2DM and is characterized by an extensive phenotyping approach. Eligible for participation were all individuals aged between 40 and 75 years and living in the southern part of the Netherlands. Participants were recruited through mass media campaigns and from the municipal registries and the regional Diabetes Patient Registry via mailings. Recruitment was stratified according to known T2DM status, with an oversampling of individuals with T2DM, for reasons of efficiency. The present report includes cross-sectional data from the first 3451 participants, who completed the baseline survey between November 2010 and September 2013. The examinations of each participant were performed within a time window of three months. The study has been approved by the institutional medical ethical committee (NL31329.068.10) and the Minister of Health, Welfare and Sports of the Netherlands (Permit 131088-105234-PG). All participants gave written informed consent. The study was performed in accordance with the Declaration of Helsinki^27^.

For the present study, individuals with self-reported history of AMI, type 1 diabetes mellitus (T1DM), or missing data on one or more of the independent variables or dependent variables were excluded. Self-reported history of AMI was examined using the Rose questionnaire^28^.

### Definition of ECG changes indicative of cardiac abnormalities

**Figure 4:**
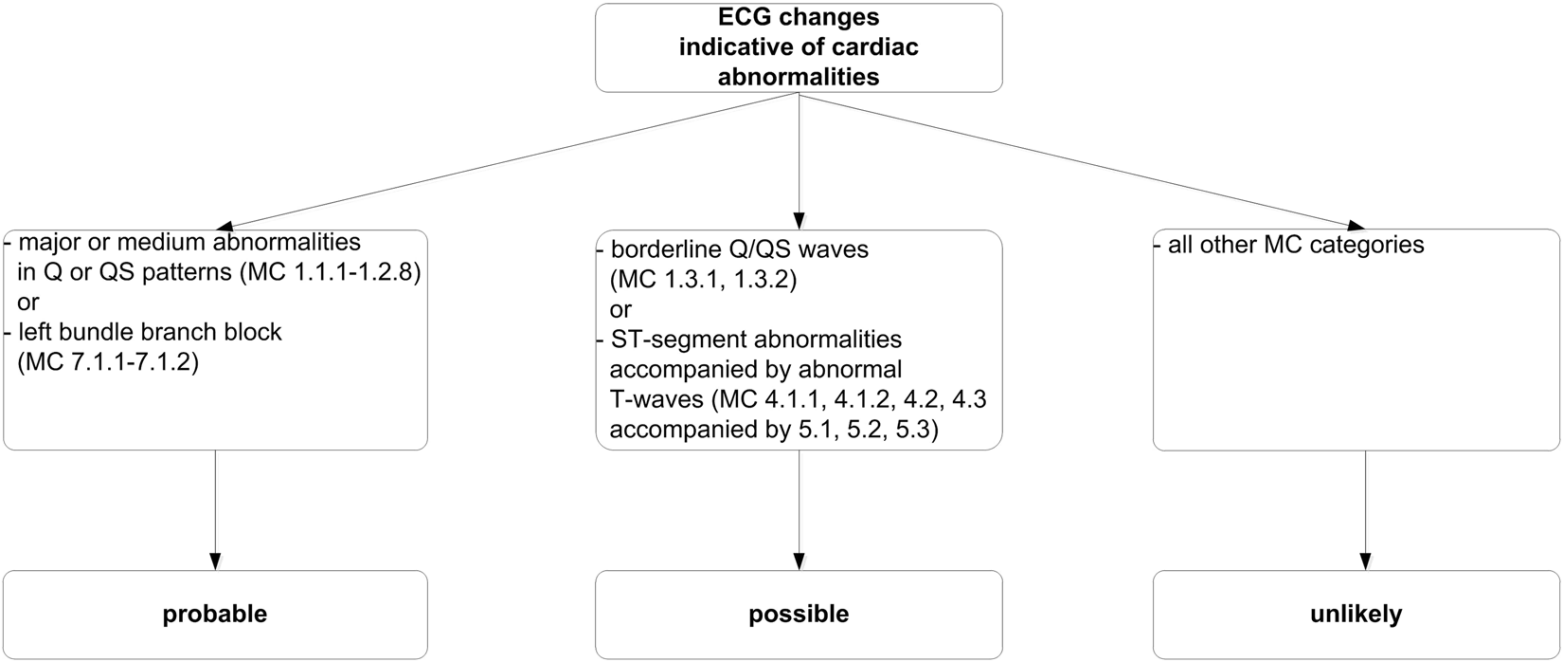
ECG changes indicative of cardiac abnormalities categorized according to Whitehall criteria into “probable”, “possible” and “unlikely”. Abbreviations: ECG, electrocardiography; MC, Minnesota Coding.

A resting 12-lead ECG was obtained using the Mac 5500 ECG system (GE Medical Systems, Milwaukee, Wisconsin, USA). ECG data were processed automatically and coded according to the Minnesota code classification system (Fysiologic ECG Services B.V., Amsterdam, the Netherlands)^29–31^. Outcomes of interests were ECG changes indicative of cardiac abnormalities, classified according to the Whitehall criteria, which are indicating the relative likelihood of signs of cardiac ischemia^32^. ECG changes indicative of cardiac abnormalities were categorized as “probable”, “possible” or “unlikely”^32^. “Probable” was attributed in case of major or medium abnormalities in Q or QS patterns or left bundle branch block (Minnesota coding; 1.1.1-1.2.8 or 7.1.1-7.1.2); “possible” was used for borderline Q/QS waves, or ST-segment abnormalities accompanied by abnormal T-waves (Minnesota coding; 1.3.1, 1.3.2 or 4.1.1, 4.1.2, 4.2, 4.3 accompanied by 5.1, 5.2, 5.3); and “unlikely” for all other Minnesota coding categories (Fig. 4).

### Biomarker measurements

Fasting blood samples were collected, centrifuged, and serum was stored in aliquots at −80 °C. Storage time prior to analyses varied from 1–4 years and was identical for hs-cTnI and hs-cTnT. Cardiac troponin I and T were measured in serum using the ARCHITECT i2000 SR analyzer (Abbott Diagnostics, Lake Forest, IL, USA) and Roche Cobas-6000 analyzer (F. Hoffman-La Roche Ltd, Basel, Switzerland), respectively. For hs-cTnI the limit of blank (LoB) ranged from 0.7 to 1.3 ng/L, the LoD ranged from 1.1 to 1.9 ng/L and the LoQ ranged from 4.0 to 10 ng/L (package insert). For hs-cTnT the LoB was 3 ng/L, the LoD was 5 ng/L and the LoQ was 13 ng/L (package insert)^33^. The hs-cTnI assay achieves a 10% CV at 4.7 ng/L and a 20% CV at 1.3 ng/L. The hs-cTnT assay achieves a 10% CV at 13 ng/L and a 20% CV at 6.8 ng/L (package insert)^33^. Cystatin C and creatinine were measured in all serum samples using the Roche Cobas-8000 analyzer (F. Hoffman-La Roche Ltd, Basel, Switzerland) and eGFR was calculated with the Chronic Kidney Disease Epidemiology Collaboration (CKD-EPI) equation based on both cystatin C and creatinine^34^.

### Potential confounders

We assessed glucose metabolism status, eGFR, smoking behavior, total-to-HDL cholesterol ratio, triglyceride levels, lipid-modifying medication, office or 24 h average ambulatory systolic blood pressure, antihypertensive medication, waist-to-hip ratio, alcohol consumption and educational level as described previously^26, 35, 36^.

### Statistical analysis

Continuous variables were expressed as mean and standard deviation (SD), or median and interquartile range (IQR) for non-Gaussian distributions. Categorical data were reported as n (%). The correlation between natural log-transformed (ln) hs-cTnI and ln hs-cTnT was assessed by Pearson’s correlation test. Cohen’s kappa (κ) was used to assess the concordance between the hs-cTnI and hs-cTnT assays, and the 95% confidence interval (CI) around κ was calculated using bias-corrected accelerated bootstrapping (resampling with replacement: 5000 bootstrap replicates). Multivariable logistic regression analyses were conducted to quantify the associations between both troponins and ECG changes indicative of cardiac abnormalities. In the primary analyses, the ECG abnormality categories “possible” and “unlikely” were combined into a single reference category. To allow for a direct comparison of troponin I and T, the independent variables hs-cTnI and hs-cTnT were tested separately in the statistical models as described below. The hs-cTn values below the LoB were set equal to the LoB value (hs-cTnI, 0.9 ng/L: hs-cTnT, 3 ng/L)/2. For hs-cTnI a LoB value of 0.9 ng/L was chosen to obtain similar proportions for troponin I and T of participants with values above the LoB. Accordingly, hs-cTnI and hs-cTnT were natural log-transformed and examined per 1-SD increase in the regression model. The independent variables (hs-cTnI, hs-cTnT) were analyzed in four different models with sequential adjustment for potential confounders; model 1: crude model; model 2: model 1+ sex, age, glucose metabolism status; model 3: model 2+ eGFR; model 4A: model 3+ smoking behavior, total-to-HDL cholesterol ratio, triglyceride levels, lipid-modifying medication, office systolic blood pressure, antihypertensive medication, waist-to-hip ratio, alcohol consumption, educational level; model 4B: model 4A with replacement of office systolic blood pressure by 24 h average ambulatory systolic blood pressure. Because of the design of The Maastricht Study, we tested for interaction between hs-cTn and glucose metabolism status. Interaction was considered significant when the *p* value (*p*_*interaction*_) of the interaction term was <0.10. No significant interaction was found for glucose metabolism status and all analyses were, therefore, conducted unstratified. In secondary analyses, we set the hs-cTn values below LoD, 20% CV, 10% CV and LoQ equally to these lower limits of measurements. Furthermore, to allow for a more liberal definition of ECG changes indicative of cardiac abnormalities, “possible” and “probable” were combined as one outcome category and compared with the reference category “unlikely”. All statistical analyses were performed using SPSS for windows 23.0 (IBM Corp., Armonk, NY, USA).

### Data Availability

The datasets generated during and/or analysed during the current study are not publicly available due to privacy issues and national laws but are available from the management board of The Maastricht Study on reasonable request under the provision that data may not leave the university/hospital premises.

## Electronic supplementary material

Supplementary data
