# Supplementary material for: Troponin I and T in relation to cardiac injury detected with electrocardiography in a population-based cohort - The Maastricht Study
Source: Sci Rep. 2017 Jul 26;7:6610. doi: 10.1038/s41598-017-06978-3 (PMC5529453; doi:10.1038/s41598-017-06978-3)
Supplement: Supplementary file 1 — Supplementary data [file 41598_2017_6978_MOESM1_ESM.pdf]

Supplementary File

Troponin I and T in relation to cardiac injury detected with electrocardiography in a population-based cohort - The Maastricht Study

Dorien M. Kimenai[1,2], Remy J.H. Martens[3,4], Jeroen P. Kooman[3,4], Coen D.A. Stehouwer[2,5,6], Frans E.S. Tan[7], Nicolaas C. Schaper[2,5], Pieter C. Dagnelie[2,6,8], Miranda T. Schram[2,5,9], Carla J.H. van der Kallen[2,5], Simone J.S. Sep[2,5], Jeroen D.E. van Suijlen[10], Abraham A. Kroon[2,5], Otto Bekers[1,2], Marja P. van Dieijen-Visser[1,2], Ronald M.A. Henry[2,5,9], Steven J.R. Meex[1,2]

[1]Department of Clinical Chemistry, Central Diagnostic Laboratory, Maastricht University Medical Center+ (MUMC+), Maastricht, the Netherlands; [2]CARIM School for Cardiovascular Diseases, Maastricht University, Maastricht, the Netherlands; [3]Department of Internal Medicine, Division of Nephrology, MUMC+, Maastricht, the Netherlands; [4]NUTRIM School of Nutrition and Translational Research in Metabolism, Maastricht University, Maastricht, the Netherlands; [5]Department of Internal Medicine, MUMC+, Maastricht, the Netherlands; [6]CAPHRI School for Public Health and Primary Care, Maastricht University, Maastricht, the Netherlands; [7]Department of Methodology and Statistics, Maastricht University, Maastricht, the Netherlands; [8]Department of Epidemiology, MUMC+, Maastricht, the Netherlands; [9]Heart and Vascular Centre, MUMC+, Maastricht, the Netherlands; [10]Department of Clinical Chemistry, Gelre Hospital, Apeldoorn, the Netherlands

Supplemental Figures

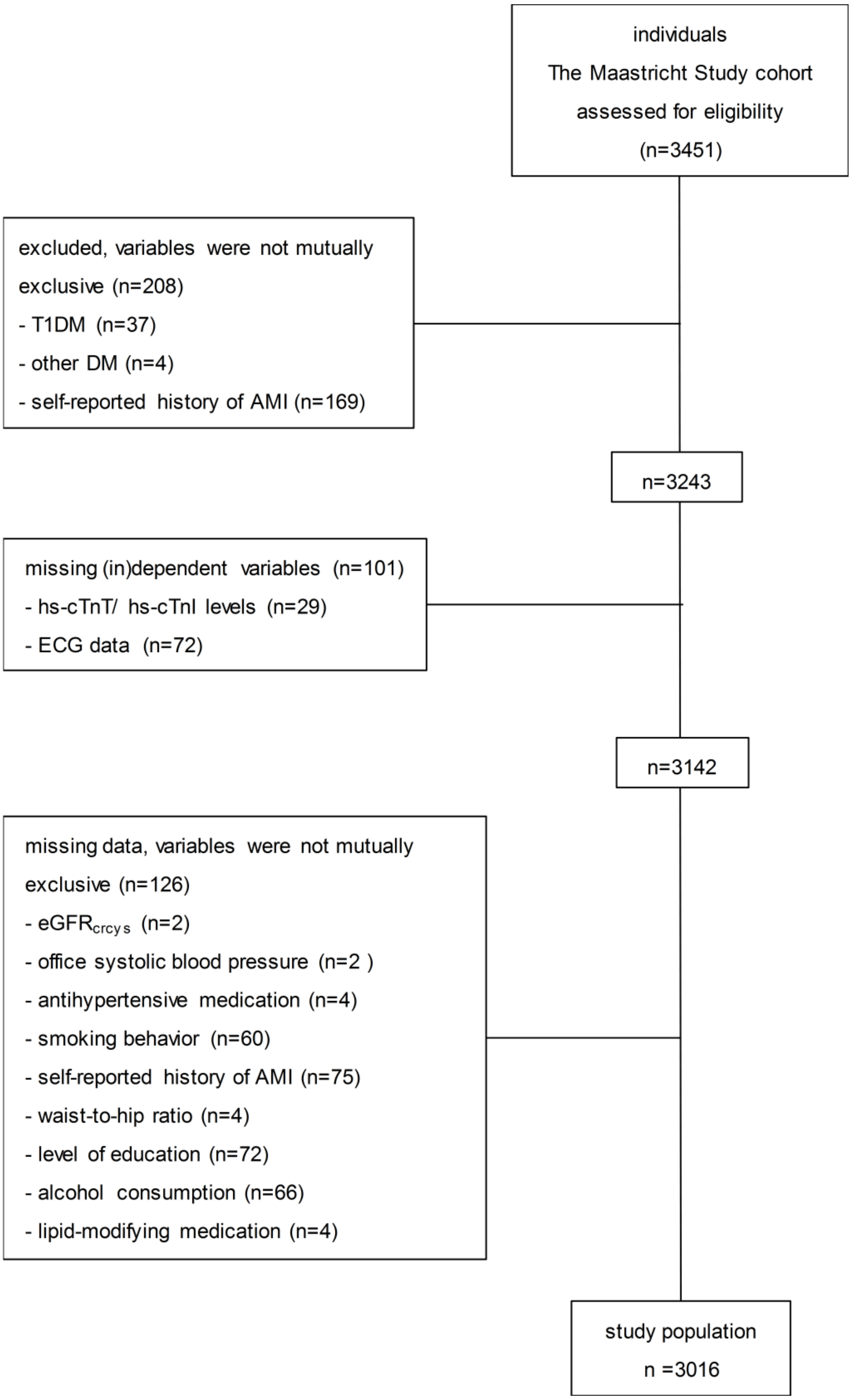

Figure S1. Study flow diagram. Abbreviations: AMI, acute myocardial infarction; DM, diabetes mellitus; eGFR, estimated Glomerular Filtration Rate; ECG, electrocardiographic; hs-cTnI, high-sensitivity cardiac troponin I; hs-cTnT, high-sensitivity cardiac troponin T; T1DM, type 1 diabetes mellitus.

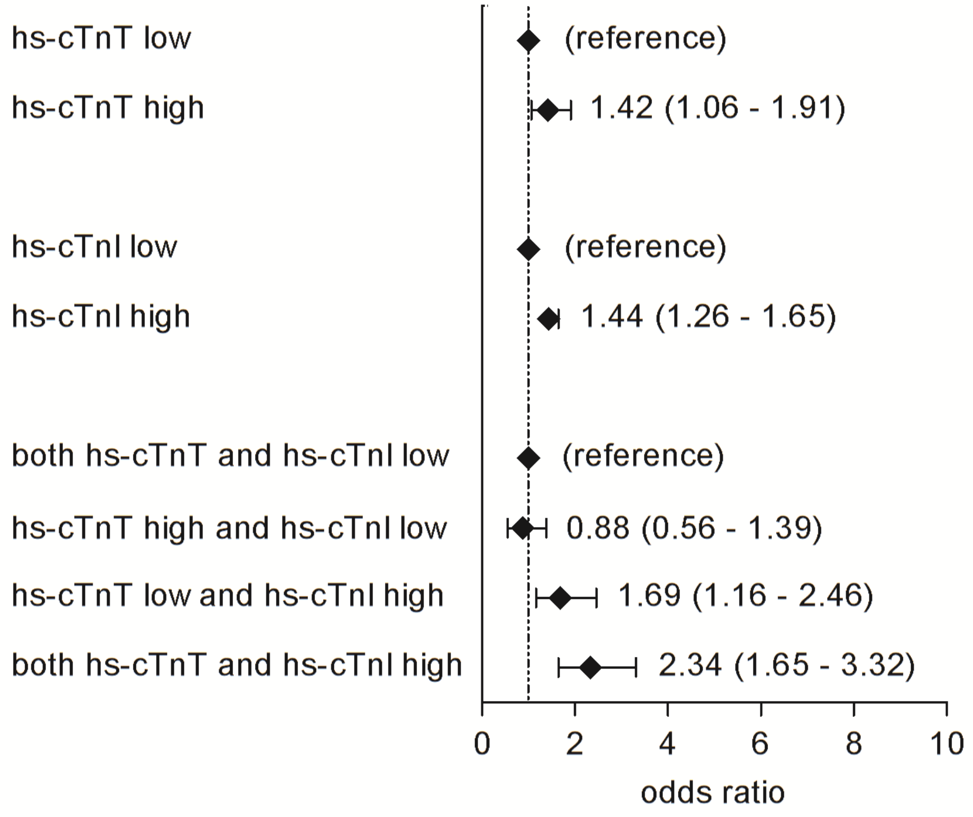

Figure S2. Associations between combined hs-cTn categories and liberal definition of ECG changes indicative of cardiac abnormalities. Definitions of "low" and "high" hs-cTn categories were based on the sex-specific 75th percentiles of hs-cTnI and hs-cTnT (hs-cTnI, women: 2.20 ng/L; hs-cTnI, men: 3.70 ng/L; hs-cTnT, women: 5.55 ng/L; hs-cTnT, men: 9.36 ng/L). Category "low" included participants with hs-cTn levels < the sex-specific 75th percentile and category "high" included participants ≥ the sex-specific 75th percentile. Model was adjusted for sex, age, glucose metabolism status, eGFR, smoking behavior, total-to-HDL cholesterol ratio, triglyceride levels, lipid-modifying medication, office systolic blood pressure, antihypertensive medication, waist-to-hip ratio, alcohol consumption and educational level. Abbreviations: ECG, electrocardiographic; eGFR, estimated Glomerular Filtration Rate; HDL, high-density lipoprotein; hs-cTnI, high-sensitivity cardiac troponin I; hs-cTnT, high-sensitivity cardiac troponin T.

Supplemental Tables

Table S1. Prevalence of Minnesota coding categories of ECG abnormalities of the total study population

Minnesota coding	description	N (%)
1.1.1 – 1.2.8	major or medium abnormalities in Q/ QS patterns	78 (2.6%)
1.3.1, 1.3.2	borderline abnormalities in Q/QS patterns	65 (2.2%)
4.1.1, 4.1.2, 4.2, 4.3	ST-segment abnormalities	82 (2.7%)
5.1, 5.2, 5.3	abnormal T-waves	310 (10.3%)
7.1.1, 7.1.2	left bundle branch block	28 (0.9%)

Data are reported as n (%)

Table S2. Associations of hs-cTnI and hs-cTnT with ECG changes indicative of cardiac abnormalities

	hs-cTnI ln, 1-SD increase		hs-cTnT ln, 1-SD increase	
	OR (95% CI)	*p*-value	OR (95% CI)	*p*-value
LoD				
model 1	1.52 (1.33-1.73)	< 0.001	1.42 (1.23-1.65)	< 0.001
model 2	1.51 (1.31-1.75)	< 0.001	1.32 (1.11-1.58)	0.002
model 3	1.54 (1.33-1.77)	< 0.001	1.38 (1.15-1.66)	0.001
model 4A	1.52 (1.31-1.76)	< 0.001	1.35 (1.11-1.63)	0.002
model 4B	1.61 (1.38-1.89)	< 0.001	1.35 (1.10-1.65)	0.004
20% CV				
model 1	1.56 (1.36-1.80)	< 0.001	1.33 (1.17-1.52)	< 0.001
model 2	1.56 (1.33-1.82)	< 0.001	1.23 (1.06-1.43)	0.007
model 3	1.58 (1.35-1.85)	< 0.001	1.27 (1.08-1.48)	0.003
model 4A	1.56 (1.32-1.83)	< 0.001	1.25 (1.06-1.47)	0.007
model 4B	1.67 (1.41-1.98)	< 0.001	1.23 (1.04-1.47)	0.019
10% CV				
model 1	1.29 (1.17-1.43)	< 0.001	1.12 (0.99-1.27)	0.069
model 2	1.30 (1.16-1.44)	< 0.001	1.06 (0.93-1.22)	0.386
model 3	1.30 (1.17-1.45)	< 0.001	1.07 (0.93-1.23)	0.325
model 4A	1.30 (1.17-1.46)	< 0.001	1.07 (0.93-1.23)	0.344
model 4B	1.34 (1.20-1.50)	< 0.001	1.06 (0.92-1.23)	0.412
LoQ				
model 1	1.24 (1.13-1.37)	< 0.001	1.12 (0.99-1.27)	0.069
model 2	1.25 (1.13-1.38)	< 0.001	1.06 (0.93-1.22)	0.386
model 3	1.26 (1.14-1.39)	< 0.001	1.07 (0.93-1.23)	0.325
model 4A	1.26 (1.13-1.39)	< 0.001	1.07 (0.93-1.23)	0.344
model 4B	1.29 (1.16-1.43)	< 0.001	1.06 (0.92-1.23)	0.412

Hs-cTn values below the lower limits of measurement were set equal to the LoD, 20% CV, 10% CV and LoQ, respectively. Hs-cTnI, LoD: 1.7 ng/L, 20% CV: 1.3 ng/L, 10% CV: 4.7 ng/L, LoQ: 6.7 ng/L; hs-cTnT, LoD: 5 ng/L, 20% CV: 6.8 ng/L, 10% CV: 13 ng/L, LoQ: 13 ng/L. For hs-cTnI a LoD value of 1.7 ng/L and LoQ value of 6.7 ng/L was chosen to obtain similar proportions for troponin I and T of participants with values above the LoD and LoQ, respectively. Model 1: crude model; model 2: model 1 + sex, age, glucose metabolism status; model 3: model 2 + eGFR; model 4A: model 3 + smoking behavior, total-to-HDL cholesterol ratio, triglyceride levels, lipid-modifying medication, office systolic blood pressure, antihypertensive medication, waist-to-hip ratio, alcohol consumption, educational level; model 4B: model 4A with replacement of office systolic blood pressure by 24h average ambulatory systolic blood pressure. Abbreviations: CV, coefficient of variation; ECG, electrocardiographic; eGFR, estimated Glomerular Filtration Rate; HDL, high-density lipoprotein; hs-cTnI, high-sensitivity cardiac troponin I; hs-cTnT, high-sensitivity cardiac troponin T; LoD, limit of detection; LoQ, limit of quantitation.

Table S3. Associations of hs-cTnI and hs-cTnT with liberal definition of ECG changes indicative of cardiac abnormalities

	hs-cTnI<br>ln, 1-SD increase		hs-cTnT<br>ln, 1-SD increase	
	OR (95% CI)	*p*-value	OR (95% CI)	*p*-value
model 1	1.54 (1.37-1.73)	< 0.001	1.40 (1.24-1.59)	< 0.001
model 2	1.52 (1.34-1.73)	< 0.001	1.33 (1.13-1.55)	< 0.001
model 3	1.52 (1.33-1.72)	< 0.001	1.32 (1.12-1.56)	0.001
model 4A	1.48 (1.30-1.69)	< 0.001	1.26 (1.06-1.49)	0.007
model 4B	1.50 (1.30-1.72)	< 0.001	1.29 (1.08-1.54)	0.006

Model 1: crude model; model 2: model 1 + sex, age, glucose metabolism status; model 3: model 2 + eGFR; model 4A: model 3 + smoking behavior, total-to-HDL cholesterol ratio, triglyceride levels, lipid-modifying medication, office systolic blood pressure, antihypertensive medication, waist-to-hip ratio, alcohol consumption, educational level; model 4B: model 4A with replacement of office systolic blood pressure by 24h average ambulatory systolic blood pressure. Abbreviations: ECG, electrocardiographic; eGFR, estimated Glomerular Filtration Rate; HDL, high-density lipoprotein; hs-cTnI, high-sensitivity cardiac troponin I; hs-cTnT, high-sensitivity cardiac troponin T.

Table S4. Clinical characteristics stratified by hs-cTnI categories based on the sex-specific 75th percentiles and sex-specific 99th percentiles

variable	hs-cTnI < sex-specific 75th percentiles (n = 2278)	hs-cTnI ≥ sex-specific 75th percentiles (n = 738)	hs-cTnI < sex-specific 99th percentiles (n = 2987)	hs-cTnI ≥ sex-specific 99th percentiles (n = 29)
age (years)	59 (8)	63 (7)	60 (8)	62 (9)
sex (male)	1151 (50.5%)	375 (50.8%)	1511 (50.6%)	15 (51.7%)
BMI (kg/m^2)	26.5 (4.3)	28.0 (4.9)	26.9 (4.5)	26.0 (3.7)
eGFR				
< 60 mL/min/1.73m^2	48 (2.1%)	61 (8.3%)	106 (3.5%)	3 (10.3%)
60 - > 90 mL/min/1.73m^2	1022 (44.9%)	416 (56.4%)	1419 (47.5%)	19 (65.5%)
≥ 90 mL/min/1.73m^2	1208 (53.0%)	261 (35.4%)	1462 (48.9%)	7 (24.1%)
glucose metabolism				
NGM	1394 (61.2%)	382 (51.8%)	1759 (58.9%)	17 (58.6%)
IFG	105 (4.6%)	24 (3.3%)	129 (4.3%)	0 (0%)
IGT	237 (10.4%)	93 (12.6%)	326 (10.9%)	4 (13.8%)
T2DM	542 (23.8%)	239 (32.4%)	773 (25.9%)	8 (27.6%)
office BP, systolic (mmHg)	133 (17)	140 (20)	135 (18)	142 (8)
office BP, diastolic (mmHg)	76 (10)	77 (11)	76 (10)	77 (11)

hypertension (yes)	1098 (48.2%)	530 (71.8%)	1609 (53.9%)	19 (65.5%)
antihypertensive medication (yes)	717 (31.5%)	391 (53.0%)	1095 (36.7%)	13 (44.8%)
smoking behavior				
never	813 (35.7%)	277 (37.5%)	1078 (36.1%)	12 (41.4%)
former	1151 (50.5%)	374 (50.7%)	1508 (50.5%)	17 (58.6%)
current	314 (13.8%)	87 (11.8%)	401 (13.4%)	0 (0%)
total-to-HDL cholesterol ratio	3.4 (2.8-4.3)	3.6 (2.8-4.3)	3.5 (2.8-4.3)	3.2 (2.8-4.3)
triglycerides (mmol/L)	1.2 (0.9-1.7)	1.3 (0.9-1.8)	1.2 (0.9-1.7)	1.0 (0.9-1.4)
lipid-modifying medication (yes)	682 (29.9%)	310 (42.0%)	984 (32.9%)	8 (27.6%)
hs-cTnI (ng/L)	1.5 (1.1-2.1)	4.3 (3.1-6.6)	1.9 (1.2-2.9)	27.9 (20.3-57.0)
hs-cTnT (ng/L)	4.8 (3.5-6.7)	7.8 (5.5-11.1)	5.3 (3.7-7.7)	11.7 (6.4-17.2)

Sex-specific 75th percentiles of hs-cTnI: women, 2.20 ng/l; men, 3.70 ng/l. Sex-specific 99th percentiles of hs-cTnI: women, 13.15 ng/l; men, 25.56 ng/l. Continuous variables are expressed as mean (SD) or median (IQR) depending on their distribution. Categorical data are reported as n (%). Abbreviations: BP, blood pressure; BMI, Body Mass Index; eGFR, estimated Glomerular Filtration Rate; HDL, high-density lipoprotein; hs-cTnI, high-sensitivity cardiac troponin I; NGM, normal glucose metabolism; IFG, impaired fasting glucose; IGT, impaired glucose tolerance; T2DM, type 2 diabetes mellitus.

Table S5. Clinical characteristics stratified by hs-cTnT categories based on the sex-specific 75th percentiles and sex-specific 99th percentiles

variable	hs-cTnT < sex-specific 75th percentiles (n = 2266)	hs-cTnT ≥ sex-specific 75th percentiles (n = 750)	hs-cTnT < sex-specific 99th percentiles (n = 2987)	hs-cTnT ≥ sex-specific 99th percentiles (n = 29)
age (years)	58 (8)	64 (7)	60 (8)	66 (7)
sex (male)	1147 (50.6%)	379 (50.5%)	1511 (50.6%)	15 (51.7%)
BMI (kg/m^2)	26.5 (4.2)	28.2 (5.1)	26.9 (4.5)	27.9 (6.1)
eGFR				
< 60 mL/min/1.73m^2	27 (1.2%)	82 (10.9%)	98 (3.3%)	11 (37.9%)
60 - > 90 mL/min/1.73m^2	958 (42.3%)	480 (64.0%)	1420 (47.5%)	18 (62.1%)
≥ 90 mL/min/1.73m^2	1281 (56.5%)	188 (25.1%)	1469 (49.2%)	0 (0%)
glucose metabolism				
NGM	1467 (64.7%)	309 (41.2%)	1769 (59.2%)	7 (24.1%)
IFG	97 (4.3%)	32 (4.3%)	129 (4.3%)	0 (0%)
IGT	239 (10.5%)	91 (12.1%)	328 (11.0%)	2 (6.9%)
T2DM	463 (20.4%)	318 (42.4%)	761 (25.5%)	20 (69.0%)
office BP, systolic (mmHg)	133 (17)	139 (19)	135 (18)	140 (23)
office BP, diastolic (mmHg)	76 (10)	76 (10)	76(10)	72 (11)

hypertension (yes)	1082 (47.7%)	546 (72.8%)	1604 (53.7%)	24 (82.8%)
antihypertensive medication (yes)	670 (29.6%)	438 (58.4%)	1087 (36.4%)	21 (72.4%)
smoking behavior				
never	821 (36.2%)	269 (35.9%)	1081 (36.2%)	9 (31.0%)
former	1133 (50.0%)	392 (52.3%)	1507 (50.5%)	18 (62.1%)
current	312 (13.8%)	89 (11.9%)	399 (13.4%)	2 (6.9%)
total-to-HDL cholesterol ratio	3.5 (2.8-4.3)	3.5 (2.8-4.3)	3.5 (2.8-4.3)	3.7 (2.7-4.3)
triglycerides (mmol/L)	1.2 (0.9-1.6)	1.3 (1.0-1.8)	1.2 (0.9-1.7)	1.4 (1.0-2.5)
lipid-modifying medication (yes)	615 (27.1%)	377 (50.3%)	980 (32.8%)	12 (41.4%)
hs-cTnI (ng/L)	1.6 (1.1-2.4)	3.0 (1.9-5.1)	1.9 (1.2-2.9)	4.6 (3.1-16.0)
hs-cTnT (ng/L)	4.5 (3.5-5.9)	10.1 (7.1-12.9)	5.3 (3.7-7.6)	33.9 (29.5-50.5)

Sex-specific 75th percentiles of hs-cTnT: women, 5.55 ng/l; men, 9.36 ng/l. Sex-specific 99th percentiles of hs-cTnT: women, 16.28 ng/l; men, 29.50 ng/l. Continuous variables are expressed as mean (SD) or median (IQR) depending on their distribution. Categorical data are reported as n (%). Abbreviations: BP, blood pressure; BMI, Body Mass Index; eGFR, estimated Glomerular Filtration Rate; HDL, high-density lipoprotein; hs-cTnT, high-sensitivity cardiac troponin T; NGM, normal glucose metabolism; IFG, impaired fasting glucose; IGT, impaired glucose tolerance; T2DM, type 2 diabetes mellitus.

Table S6. Associations between combined hs-cTn categories and ECG changes indicative of cardiac abnormalities

threshold	hs-cTn categories	n	OR (95% CI)	*p*-value
66th percentile	both hs-cTnT and hs-cTnI low	1526	reference	
	hs-cTnT high and hs-cTnI low	381	0.98 (0.43-2.22)	0.953
	hs-cTnT low and hs-cTnI high	444	1.67 (0.86-3.25)	0.129
	both hs-cTnT and hs-cTnI high	625	3.32 (1.91-5.77)	< 0.001
70th percentile	both hs-cTnT and hs-cTnI low	1692	reference	
	hs-cTnT high and hs-cTnI low	360	1.02 (0.46-2.30)	0.955
	hs-cTnT low and hs-cTnI high	418	1.74 (0.90-3.34)	0.098
	both hs-cTnT and hs-cTnI high	546	3.57 (2.07-6.16)	< 0.001
80th percentile	both hs-cTnT and hs-cTnI low	2093	reference	
	hs-cTnT high and hs-cTnI low	296	1.24 (0.58-2.68)	0.577
	hs-cTnT low and hs-cTnI high	320	2.35 (1.27-4.34)	0.006
	both hs-cTnT and hs-cTnI high	370	3.92 (2.20-6.97)	< 0.001
90th percentile	both hs-cTnT and hs-cTnI low	2524	reference	
	hs-cTnT high and hs-cTnI low	182	1.34 (0.60-2.96)	0.477
	hs-cTnT low and hs-cTnI high	191	2.53 (1.34-4.80)	0.004
	both hs-cTnT and hs-cTnI high	119	3.83 (1.90-7.72)	< 0.001

Definitions of “low” and “high” hs-cTn categories were based on sex-specific 66th percentiles of hs-cTnI and hs-cTnT (hs-cTnI, women: 1.80 ng/L; hs-cTnI, men: 3.10 ng/L; hs-cTnT, women: 4.95 ng/L; hs-cTnT, men: 8.34 ng/L), 70th percentiles (hs-cTnI, women: 1.90 ng/L; hs-cTnI, men: 3.30 ng/L; hs-cTnT, women: 5.18 ng/L; hs-cTnT, men: 8.68 ng/L), 80th percentiles (hs-cTnI, women: 2.40 ng/L; hs-cTnI, men: 4.00 ng/L; hs-cTnT, women: 5.93 ng/L; hs-cTnT, men: 10.17 ng/L) and 90th percentiles (hs-cTnT, women: 7.68 ng/L; hs-cTnI, women: 3.50 ng/L; hs-cTnI, men: 6.30 ng/L; hs-cTnT, men: 12.83 ng/L). Category “low” included participants with hs-cTn levels < the respective percentile and category “high” included participants ≥ the respective percentile. Model was adjusted for sex, age, glucose metabolism status, eGFR, smoking behavior, total-to-HDL cholesterol ratio, triglyceride levels, lipid-modifying medication, office systolic blood pressure, antihypertensive medication, waist-to-hip ratio, alcohol

consumption and educational level. Abbreviations: ECG, electrocardiographic; eGFR, estimated Glomerular Filtration Rate; HDL, high-density lipoprotein; hs-cTnI, high-sensitivity cardiac troponin I; hs-cTnT, high-sensitivity cardiac troponin T.

Table S7. Clinical characteristics stratified by combined hs-cTn categories based on the sex-specific 75th percentiles

variable	hs-cTnT and hs-cTnI low (n = 1934)	hs-cTnT high and hs-cTnI low (n = 344)	hs-cTnT low and hs-cTnI high (n = 332)	hs-cTnT and hs-cTnI high (n = 406)
age (years)	58 (8)	63 (7)	60 (8)	65 (7)
sex (male)	979 (50.6%)	172 (50.0%)	168 (50.6%)	207 (51.0%)
BMI (kg/m^2)	26.3 (4.1)	27.7 (4.8)	27.3 (4.4)	28.7 (5.3)
eGFR				
< 60 $mL/min/1.73m^2$	22 (1.1%)	26 (7.6%)	5 (1.5%)	56 (13.8%)
60 - > 90 $mL/min/1.73m^2$	809 (41.8%)	213 (61.9%)	149 (44.9%)	267 (65.8%)
≥ 90 $mL/min/1.73m^2$	1103 (57.0%)	105 (30.5%)	178 (53.6%)	83 (20.4%)
glucose metabolism				
NGM	1253 (64.8%)	141 (41.0%)	214 (64.5%)	168 (41.4%)
IFG	90 (4.7%)	15 (4.4%)	7 (2.1%)	17 (4.2%)
IGT	196 (10.1%)	41 (11.9%)	43 (13.0%)	50 (12.3%)
T2DM	395 (20.4%)	147 (42.7%)	68 (20.5%)	171 (42.1%)
office BP, systolic (mmHg)	132 (17)	136 (17)	139 (19)	142 (20)
office BP, diastolic (mmHg)	76 (10)	76 (10)	78 (11)	76 (11)
hypertension (yes)	870 (45.0%)	228 (66.3%)	212 (63.9%)	318 (78.3%)
antihypertensive medication (yes)	531 (27.5%)	186 (54.1%)	139 (41.9%)	252 (62.1%)

smoking behavior				
never	704 (36.4%)	109 (31.7%)	117 (35.2%)	160 (39.4%)
former	959 (49.6%)	192 (55.8%)	174 (52.4%)	200 (49.3%)
current	271 (14.0%)	43 (12.5%)	41 (12.3%)	46 (11.3%)
total-to-HDL cholesterol ratio	3.4 (2.8-4.3)	3.4 (2.8-4.3)	3.6 (3.0-4.3)	3.5 (2.8-4.3)
triglycerides (mmol/L)	1.2 (0.9-1.6)	1.3 (1.0-1.8)	1.2 (0.9-1.7)	1.3 (1.0-1.9)
lipid-modifying medication (yes)	511 (26.4%)	171 (49.7%)	104 (31.3%)	206 (50.7%)
hs-cTnI (ng/L)	1.5 (1.1-2.0)	1.9 (1.4-2.5)	4.0 (2.9-5.5)	4.7 (3.5-7.4)
hs-cTnT (ng/L)	4.3 (3.2-5.7)	9.8 (6.6-11.9)	5.3 (4.3-7.4)	10.6 (7.8-14.0)

Definitions of “low” and “high” hs-cTn categories were based on the sex-specific 75th percentiles of hs-cTnI and hs-cTnT (hs-cTnI, women: 2.20 ng/L; hs-cTnI, men: 3.70 ng/L; hs-cTnT, women: 5.55 ng/L; hs-cTnT, men: 9.36 ng/L). Category “low” included participants with hs-cTn levels < the sex-specific 75th percentile and category “high” included participants ≥ the sex-specific 75th percentile. Continuous variables are expressed as mean (SD) or median (IQR) depending on their distribution. Categorical data are reported as n (%). Abbreviations: BP, blood pressure; BMI, Body Mass Index; eGFR, estimated Glomerular Filtration Rate; HDL, high-density lipoprotein; hs-cTnI, high-sensitivity cardiac troponin I; hs-cTnT, high-sensitivity cardiac troponin T; NGM, normal glucose metabolism; IFG, impaired fasting glucose; IGT, impaired glucose tolerance; T2DM, type 2 diabetes mellitus.

Table S8. Characteristics and main findings of studies investigating hs-cTnI and hs-cTnT in relation to clinical outcome under non-acute conditions

publication	time period	population	total no included	sex (male)	correlation or concordance hs-cTnI/hs-cTnT	outcome
Omland 2013[19]	1996-2000, median follow-up 5.2 years	low-risk patients with stable CAD	3623	2933 (81.0%)	r = 0.44	CVD mortality or heart failure, nonfatal AMI
Hijazi 2015[20]	2006-2010, median follow-up 1.9 years	AF patients	14806	9517 (64.3%)	r = 0.70	stroke or systemic embolism, all-cause mortality, CVD mortality, AMI, major bleeding
deFilippi 2012[21]	2006-2007, median follow-up 4.8 years	patients with stable CKD	148	102 (68.9%)	κ = 0.40	CAC, LVMI

Table S8 (continued)

publication	main findings	
Omland 2013[19]	**outcome and groups***	**HR (95% CI)**
	**CVD mortality or heart failure**	
	both hs-cTnT and hs-cTnI low (reference category)	
	(hs-cTnT high and hs-cTnI low) or (hs-cTnT high and hs-cTnI low)	2.25 (1.18-4.28)
	hs-cTnT and hs-cTnI high	7.57 (4.30-13.35)
	**non-fatal AMI**	
	both hs-cTnT and hs-cTnI low (reference category)	
	(hs-cTnT high and hs-cTnI low) or (hs-cTnT high and hs-cTnI low)	0.96 (0.66-1.39)
	hs-cTnT and hs-cTnI high	1.10 (0.79-1.55)
Hijazi 2015[20]	**outcome and groups****	**HR (95% CI)**
	**stroke or systemic embolism**	
	both hs-cTnT and hs-cTnI low (reference category)	
	hs-cTnT high and hs-cTnI low	1.35 (0.93-1.97)
	hs-cTnT high and hs-cTnI low	1.21 (0.83-1.74)
	hs-cTnT and hs-cTnI high	1.72 (1.31-2.27)

Table S8 (continued)

publication	main findings	
Hijazi 2015[20]	**outcome and groups****	**HR (95% CI)**
	**all-cause mortality**	
	both hs-cTnT and hs-cTnI low (reference category)	
	hs-cTnT high and hs-cTnI low	1.19 (0.90-1.57)
	hs-cTnT high and hs-cTnI low	1.51 (1.17-1.95)
	hs-cTnT and hs-cTnI high	2.32 (1.91-2.80)
	**CVD mortality**	
	both hs-cTnT and hs-cTnI low (reference category)	
	hs-cTnT high and hs-cTnI low	1.28 (0.83-1.98)
	hs-cTnT high and hs-cTnI low	1.97 (1.36-2.84)
	hs-cTnT and hs-cTnI high	3.14 (2.32-4.20)
	**AMI**	
	both hs-cTnT and hs-cTnI low (reference category)	
	hs-cTnT high and hs-cTnI low	1.58 (0.79-3.15)
	hs-cTnT high and hs-cTnI low	2.77 (1.48-5.16)
	hs-cTnT and hs-cTnI high	2.99 (1.75-5.03)

Table S8 (continued)

publication	main findings	
Hijazi 2015[20]	**outcome and groups****	**HR (95% CI)**
	**ischemic stroke/systemic embolism/AMI/ CVD mortality**	
	both hs-cTnT and hs-cTnI low (reference category)	
	hs-cTnT high and hs-cTnI low	1.21 (0.90-1.64)
	hs-cTnT high and hs-cTnI low	1.68 (1.29-2.18)
	hs-cTnT and hs-cTnI high	2.50 (2.04-3.07)
	**major bleeding**	
	both hs-cTnT and hs-cTnI low (reference category)	
	hs-cTnT high and hs-cTnI low	1.69 (1.30-2.18)
	hs-cTnT high and hs-cTnI low	1.41 (1.06-1.87)
	hs-cTnT and hs-cTnI high	1.54 (1.24-1.91)
deFilippi 2012[21]	**outcome and hs-cTn assay**	**risk-factor adjusted β (*p-value*)**
	**CAC**	
	hs-cTnI (continuous)	-0.15 (p = 0.09)
	hs-cTnT (continuous)	-0.15 (p = 0.15)

Table S8 (continued)

publication	main findings	
deFilippi 2012[21]	**outcome and hs-cTn assay**	**risk-factor adjusted β (*p-value)***
	**LVMI**	
	hs-cTnI (continuous)	0.36 ($p < 0.001$)
	hs-cTnT (continuous)	0.36 ($p = 0.004$)

* Definitions of “low” and “high” hs-cTn categories were based on the sex-specific 75th percentiles of hs-cTnI and hs-cTnT (hs-cTnI, women: 2.8 ng/L; hs-cTnI, men: 4.2 ng/L; hs-cTnT, women: 3.9 ng/L; hs-cTnT, men: 5.6 ng/L). ** Definitions of “low” and “high” hs-cTn categories were based on the median of hs-cTnI and hs-cTnT (hs-cTnI: 6.3 ng/L ; hs-cTnT; 17.0 ng/L). Abbreviations: AF, atrial fibrillation; AMI, acute myocardial infarction; CAC, coronary artery calcification; CAD, coronary artery disease; CKD, chronic kidney disease; CVD, cardiovascular disease; hs-cTnI, high-sensitivity cardiac troponin I; hs-cTnT, high-sensitivity cardiac troponin T; HR, hazard ratio; LVMI, left ventricular mass index.
